# Efficacy of 8- and 4-Session Mindfulness-Based Interventions in a Non-clinical Population: A Controlled Study

**DOI:** 10.3389/fpsyg.2017.01343

**Published:** 2017-08-08

**Authors:** Marcelo Demarzo, Jesus Montero-Marin, Marta Puebla-Guedea, Mayte Navarro-Gil, Paola Herrera-Mercadal, Sergio Moreno-González, Sandra Calvo-Carrión, Laura Bafaluy-Franch, Javier Garcia-Campayo

**Affiliations:** ^1^Mente Aberta – Brazilian Center for Mindfulness and Health Promotion, Universidade Federal de Sao Paulo Sao Paulo, Brazil; ^2^Hospital Israelita Albert Einstein Sao Paulo, Brazil; ^3^Faculty of Health Sciences and Sports, University of Zaragoza Huesca, Spain; ^4^Primary Care Prevention and Health Promotion Research Network (RedIAPP), Centro de Investigación Biomédica en Red de Salud Mental Zaragoza, Spain; ^5^Faculty of Health Sciences, Universidad San Jorge Zaragoza, Spain; ^6^Miguel Servet Hospital, University of Zaragoza Zaragoza, Spain

**Keywords:** mindfulness, mindfulness-based interventions, MBI, abbreviated programmes, well-being

## Abstract

**Background:** Many attempts have been made to abbreviate mindfulness programmes in order to make them more accessible for general and clinical populations while maintaining their therapeutic components and efficacy. The aim of this study was to assess the efficacy of an 8-week mindfulness-based intervention (MBI) programme and a 4-week abbreviated version for the improvement of well-being in a non-clinical population.

**Method:** A quasi-experimental, controlled, pilot study was conducted with pre-post and 6-month follow-up measurements and three study conditions (8- and 4-session MBI programmes and a matched no-treatment control group, with a sample of 48, 46, and 47 participants in each condition, respectively). Undergraduate students were recruited, and mindfulness, positive and negative affect, self-compassion, resilience, anxiety, and depression were assessed. Mixed-effects multi-level analyses for repeated measures were performed.

**Results:** The intervention groups showed significant improvements compared to controls in mindfulness and positive affect at the 2- and 6-month follow-ups, with no differences between 8- vs. 4-session programmes. The only difference between the abbreviated MBI vs. the standard MBI was found in self-kindness at 6 months, favoring the standard MBI. There were marginal differences in anxiety between the controls vs. the abbreviated MBI, but there were differences between the controls vs. the standard MBI at 2- and 6-months, with higher levels in the controls. There were no differences in depression between the controls vs. the abbreviated MBI, but differences were found between the controls vs. the standard MBI at 2- and 6-months, favoring the standard MBI. There were no differences with regard to negative affect and resilience.

**Conclusion:** To our knowledge, this is the first study to directly investigate the efficacy of a standard 8-week MBI and a 4-week abbreviated protocol in the same population. Based on our findings, both programmes performed better than controls, with similar effect size (ES). The efficacy of abbreviated mindfulness programmes may be similar to that of a standard MBI programme, making them potentially more accessible for a larger number of populations. Nevertheless, further studies with more powerful designs to compare the non-inferiority of the abbreviated protocol and addressing clinical populations are warranted.

**Clinical Trials.gov Registration ID:** NCT02643927

## Introduction

Mindfulness has been described as a mental state or trait (dispositional mindfulness) that cultivates both focused attention on and awareness of moment-by-moment experience with a non-judgemental attitude of curiosity, openness, and acceptance (Bishop et al., 2004). Thus, in a mindful state, one becomes an observer of one's own stream of consciousness, generally called a meta- or self-awareness state, which may prevent the attention and cognitive biases linked with several mental and physical disorders (Waszczuk et al., 2015). The most widely known structured programme for mindfulness training is Mindfulness-Based Stress Reduction (MBSR), an original programme created in the United States in the late 1970's (Kabat-Zinn, 1982; Kabat-Zinn et al., 1985, 2016), which has inspired the development of a number of mindfulness-based interventions (MBIs) worldwide, such as, Mindfulness-based Cognitive Therapy (MBCT; Teasdale et al., 2000). Since that time, the practice of mindfulness for the purposes of both prevention and therapy in medicine and psychology, and for use in education, business and sport, has grown exponentially. In fact, mindfulness and its many applications in society have actually become one of the most widely studied fields in neuroscience research (Tang et al., 2015).

MBSR and most of its derivations are made up of 8 weekly sessions with a duration of 120–150 min, plus a voluntary one-day or half-day retreat; therefore, they require a high level of commitment from trainees to adhere to the programme sessions and, more importantly, to the recommended 20–45 min of mindfulness practice per day (Cullen, 2011). The populations for which these programmes were first developed consisted mainly of high-income, educated people, mostly with a chronic clinical condition, who were generally more motivated and committed to the prescribed programme (Cullen, 2011; Palta et al., 2012; Amaro et al., 2014). Mindfulness is currently in wide use, and general, non-clinical populations frequently consider these programmes. Consequences of this wide popularity are now being seen in decreasing compliance with the programme sessions and daily practice, and in increasing rates of withdrawal. A possible explanation might be that courses not designed for clinical populations seem to be used by people with a significant mental health burden (Lyssenko et al., 2015), which may reduce their efficacy. On the other hand, the general population may adhere less to programmes designed for a clinical population. Moreover, it has been said that levels of alliance between participants and mindfulness instructors predict treatment compliance (Goldberg et al., 2013). Therefore, another reason for withdrawal might be a poorly established alliance between them. National healthcare systems (NHSs), such as, those found in many Western European countries, require effective, shorter, and lower-cost interventions to improve their accessibility and incorporate them into their list of evidence-based therapies. Mindfulness interventions might be offered by NHSs if we are able to achieve cost-effective programmes adjusted to the different target populations (Demarzo et al., 2015).

Many attempts have been made to shorten mindfulness programmes to make them more feasible and accessible for general and clinical populations while maintaining their therapeutic components and efficacy, adapting them to the specific target populations and contexts. For instance, variations can be found in the number of sessions (ranging from 4 to 7 sessions), the length of the sessions (from 1 to 2-h sessions), and in the presence and length of the all-day session (ranging from not available to a 6-h all-day session), always taking as reference the 2.5-h, 8-session standard from MBSR, including a 1-day, all-day session of 8 h (Carmody and Baer, 2009; Klatt et al., 2009; Fortney et al., 2013; Morrison et al., 2013; Bergen-Cico et al., 2014; Braden et al., 2016; Schroeder et al., 2016). Some of these modified programmes have had their efficacy assessed in different populations, such as, patients and professionals (Fortney et al., 2013; Bergen-Cico et al., 2014; Braden et al., 2016; Schroeder et al., 2016), young workers (Klatt et al., 2009), and students (Morrison et al., 2013). Although a comprehensive narrative review by Carmody and Baer (2009) suggested that the number of in-class hours was non-significant for both clinical and non-clinical samples to improve psychological distress, there is no meta-analysis on the efficacy of these shorter interventions, nor is there, to our knowledge, a comparison between standardized protocols, such as, MBSR and these abbreviated programmes.

The aim of this study was to assess the efficacy of a standard 8-week protocol inspired by the MBSR programme and the efficacy of a 4-week abbreviated version of the protocol for the improvement of mindfulness and of psychological well-being variables in a non-clinical population. Our initial hypothesis was that both protocols would be efficacious for the improvement of mindfulness. As a secondary hypothesis, we considered that both protocols would also be efficacious for the improvement of psychological well-being.

## Methods

### Design

A quasi-experimental, controlled, 6-month follow-up pilot study was conducted with pre-post and follow-up measurements, and three study conditions. The study conditions were as follows: (1) eight standard 2-h sessions of an MBI, (2) an abbreviated version of the referred MBI with four 2-h sessions, and (3) controls who received no intervention at all. A random assignment was performed within the intervention groups (1 and 2) but not with the control group, for which a matched and purposive sample with no intervention was used (more details regarding procedures are given below).

### Participants

Undergraduate students were recruited from the Faculty of Health Sciences of the Universidad San Jorge, Zaragoza, Spain. Our sample size calculation was based on testing differences between the 8-week MBI and controls. We expected a difference in the Mindful Attention Awareness Scale (MAAS) measure of 0.6 standard deviations (Khoury et al., 2015). To detect this difference between groups, assuming equal variances, a 95% confidence level and a statistical power of 80%, 45 subjects were required for each group. As a drop-out rate of 10% was expected, the numbers were inflated to reach a total sample size of 150 subjects (50 per group).

- The inclusion criteria were (a) adults (aged 18 years or older), (b) willingness to participate in the study and to give signed informed consent, and (c) ability to understand and write in Spanish.
- The exclusion criteria were not fulfilling any of the inclusion criteria, presence of an acute clinical or psychiatric condition (checked by means of a pre-participation questionnaire and further clinical interview if considered necessary), and previous experience with mindfulness or another type of contemplative or mind-body practices such as, yoga, tai chi or qigong in the previous 6 months.

### Interventions

The three groups and respective interventions provided in this study were as follows:

- 8-Week MBI (see Table 1 for the practices introduced in each session): 8 weekly 120-min sessions were held inspired by the standard protocol by Kabat-Zinn (Kabat-Zinn, 1990; Davidson et al., 2003). The adapted programme did not include an all-day retreat session, but it did contain one full session in silence, plus one extra kindly awareness practice (a compassion-based practice addressing loving kindness and equanimity, adapted from the Breath-works CIC programme, Manchester, UK; Burch, 2008) and a values-based exercise (the “funeral exercise” from Acceptance and Commitment Therapy—ACT; Hayes and Strosahl, 2005). An enactment exercise (“1st and 2nd suffering,” from the Breath-works CIC programme (Burch, 2008), which is based on the Buddhist concept of “The First and Second Darts of Suffering”) was also used to introduce some insight into how mindfulness works. Home practice was not systematically assessed, but 45 min per day was recommended on most of the days. The programme was administered by a certified mindfulness teacher (MD) with 3 years' experience after professional training and more than 300 in-class hours of teaching in that time. Treatment integrity was checked through video recordings of the sessions by JGC, another mindfulness teacher with long-term experience.
- Abbreviated 4-week MBI (see Table 1 for the practices introduced in each session): This protocol consisted of 4 weekly 120-min sessions administered by the same trained teacher (MD) to avoid variables associated with the therapist. The content of the abbreviated 4-session intervention is given in Table 1. The rationale behind the abbreviated MBI was to keep the core content and practices of the programme over fewer sessions, which consisted of the raisin exercise (experiential concept of mindfulness) and the main anchors of attention (breathing, body sensations, and body movements through walking meditation). Unlike the 8-week programme, it did not include a standing and lying session of mindful movements, kindly awareness, a full session in silence, or a values-based exercise. In addition, the sitting meditation practice (open monitoring) was not formally introduced. Treatment integrity was also checked through video recordings of the sessions by JGC.
- Control group: No intervention. Participants only received a 1-h informative session and were invited to complete the questionnaires on psychometric data, but they did not receive any kind of intervention and were asked not to be involved in the practice of any mindfulness, meditation or body-mind technique during the study period.

**Table 1:** General programme content and main differences between 8- vs. 4-week programmes (main mindfulness practices and psycho-education introduced per session).

	**8-Week programme**	**4-Week programme**
Session 1	- Raisin exercise - What is mindfulness?	- Raisin exercise - What is mindfulness?
Session 2	- Mindfulness of breathing (MoB) - Handling thoughts and emotions during practices	- Mindfulness of breathing - Handling thoughts and emotions during practices
Session 3	- Body scan (BS) - 1st and 2nd suffering	- Body scan - 1st and 2nd suffering
Session 4	- Walking meditation (WM)	- Walking meditation - Practice review - Incorporating mindfulness in daily life - End of the programme
Session 5	- Mindful movements (MM), sitting meditation	No
Session 6	- Full session in silence (sequence of sitting meditation + BS + WM + MM)	No
Session 7	- Kindly awareness	No
Session 8	- Value-based practice - Practice review - Incorporating mindfulness in daily life - End of the programme	No

### Measurements

Pre- (baseline, 1 week before interventions), post- (2 months after baseline, 1 week after the end of the longer intervention), and 6-month follow-up (after post-test) measurements were administered. All the groups followed the same timetable. The following instruments were administered to the three study conditions:

- Socio-demographics*:* A set of data was recorded including gender, age, marital status and prior meditative experience (previous 6 months).
- Main variable: MAAS (Brown and Ryan, 2003) was considered the main measure. It is a 15-item unidimensional measure of mindfulness. Each item is rated on a Likert scale between 1 (almost always) and 6 (almost never) in relation to the respondent's everyday experience (e.g., “I rush through activities without being really attentive to them”). Higher scores reflect higher levels of dispositional mindfulness. The scale has been validated in Spanish with appropriate psychometric features (Soler et al., 2012). In the protocol registration, it was stated that the primary outcome was anxiety/depression. However, prior to beginning the study, it was determined to change the primary outcome to a mindfulness measure, because it was supposed that treatment with a healthy population might make the observation of possible differences in the groups' functioning difficult due to possible floor effects in clinical variables. Both treatments were primarily based on an MBI; therefore, mindfulness was finally considered the main target.
- Secondary variables: Based on previous studies in non-clinical populations, we chose a large number of secondary measures as proxies for well-being that are supposed to be sensitive to MBIs. These comprised mindfulness and its dimensions (Mak et al., 2015), self-compassion (Greeson et al., 2014; Lever Taylor et al., 2014), positive and negative affect (Gambrel and Piercy, 2015), anxiety and depressive symptoms, and resilience (Lyssenko et al., 2015).

Five Facets Mindfulness Questionnaire (FFMQ; Baer et al., 2006): This measure consists of 39 items that assess five facets of mindfulness. Items are rated on a Likert scale ranging between 1 (never or very rarely true) and 5 (very often or always true), with higher scores representing higher self-reported mindfulness skills. The five facets are observing, describing, acting with awareness, non-judging, and non-reactivity to inner experience. The Spanish version of the FFMQ has been validated and shows good psychometric properties (Cebolla et al., 2012).

Self-Compassion Scale (SCS): SCS (Neff, 2003a) is a 26-item questionnaire designed to assess self-compassion across the facets of common humanity, mindfulness and self-kindness. Although the construct was defined using these three facets (Neff, 2003b), factor analysis suggests six subscales representing the positive and negative aspects of each facet. The items assess how respondents perceive their actions toward themselves at times of difficulty and are rated using a Likert-type scale anchored between 1 (almost never) and 5 (almost always). The SCS has shown adequate properties, even in different cultures (Neff et al., 2008). The Spanish version (Garcia-Campayo et al., 2014) of the SCS was used.

The Positive and Negative Affect Schedule (PANAS): The PANAS (Watson et al., 1988) is a self-report instrument that measures positive and negative affect. This questionnaire consists of a list of 20 adjectives, 10 per subscale (e.g., positive: “interested”; e.g., negative: “guilty”), rated on a 5-point scale, and using the time instructions desired by the researcher (for the present study we used the previous week). This questionnaire has been validated in Spanish with good psychometric properties (Sandín et al., 1999).

Hospital Anxiety and Depression Scale (HADS): HADS assesses (possible or probable) cases of anxiety and depression in a non-psychiatric population. This scale is divided into the anxiety subscale (HADS-A, with seven items) and the depression subscale (HADS-D, with seven items; Bjelland et al., 2002). We used the Spanish validated version, which shows adequate psychometric characteristics (Herrero et al., 2003).

Resilience: This was evaluated using the 10-item Connor-Davidson Resilience Scale (CD-RISC; Campbell-Sills and Campbell-Sills and Stein, 2007), a self-administered questionnaire of 10 items designed as a Likert-type additive scale with five response options (between 0 = never and 4 = almost always), which had a single dimension in the original version. The final score on the questionnaire was the sum of the responses obtained for each item (range 0–40), and the highest scores indicated the highest level of resilience. We used the Spanish validated version (Notario-Pacheco et al., 2011).

### Procedures and random assignment to interventions

Students were made aware of the study by notices in the university's digital and print media. With an interval of enrolment of 2 months (December 2012, January 2013), students who volunteered to participate in the study (*n* = 1 09) were randomly allocated to one of the two intervention groups (8-week MBI or abbreviated 4-session MBI) to avoid any treatment preference bias that might influence treatment outcomes. Student volunteers (*n* = 49) who did not want to receive training were allocated to the control group. Students who voluntarily agreed to participate and complete the full protocol measurements (regardless of the study condition) were certified with a plus 0.5 credit to be applied their final grade for the semester.

The study and interventions were explained during a 1-h introductory session in the different departments where the study would be implemented. The students interested in participating were included in the study upon signing informed consent. An independent researcher who was not involved in this study randomized the students who agreed to participate, using a computer-generated randomized number list. Non-intervention group participants were offered the opportunity to take either of the two active interventions at the end of the study (after follow-up measures). As previously stated, both active interventions were delivered by the same certified mindfulness teacher to avoid any potential influence from therapist experience on the results (Crane et al., 2013). Two independent researchers (MPG, MNG, and PHM), who were blind to which group the participants had been allocated, administered the questionnaires. The mindfulness programmes were held between February and April 2013. The study was approved by the corresponding regional Ethical Board of Aragon, Spain. In addition, the study protocol was registered on the *Clinical Trials.gov* database (Registration ID: NCT02643927).

### Statistical analysis

The socio-demographic characteristics of the three groups were compared first, in order to verify that there were no significant differences between them at baseline. We used means and standard deviations for describing the continuous variables, and frequencies and percentages for the categorical variables, and groups were compared at baseline by using the corresponding χ^2^ and one-way ANOVA tests. The between-group analyses were performed on an intention-to-treat basis for the total scores of the outcomes, using hierarchical mixed-effect models by restricted maximum likelihood regression (REML), in which time acts as an independent variable, and the random part is formed by subjects. REML is less biased for the estimation of variance parameters when using small sample sizes or unbalanced data (Egbewale et al., 2014). Regression coefficients (B) and their 95% confidence interval (95% CI) were calculated for the interaction group × time, and we also reported effect sizes (ESs) by means of Cohen's *d* for each pairwise comparison. The rule of thumb for Cohen's *d* is that 0.20 is small, 0.50 is medium, and 0.80 is large.

The overall α level was set at 0.05, using Bonferroni's criterion for the primary outcome but not for the secondary outcomes, to balance between type I and type II error (Feise, 2002). All analyses were performed using the STATA v12 statistical package.

## Results

**Figure 1:**
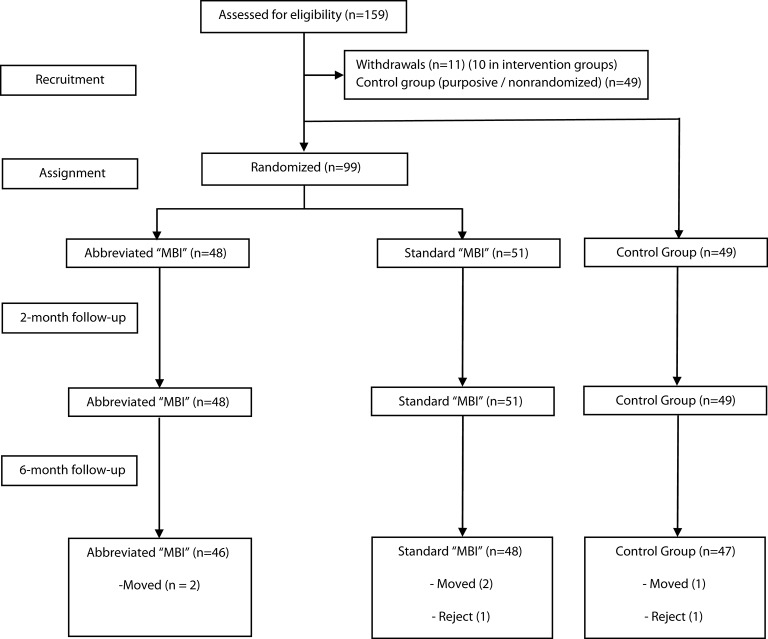
Study flowchart.

There were no exclusions resulting from non-compliance with inclusion and exclusion criteria. Of the 159 students assessed for eligibility, 11 (6.9%) withdrew from the study after the introductory session, 10 from the intervention groups, and one from the control group, finally resulting in a total of 148 participants (see Figure 1 for a flowchart of the study). Outcome data were obtained for all the participants at post-test, and for 141 participants (95.3%) at the 6-month follow-up, with no significant differences between groups in terms of attrition [controls = 4.1%; abbreviated MBI = 4.2%; 8-week MBI = 5.9%; $\chi_{\left(2\right)}^{2}=$ 0.06, *p* = 0.97]. Missing data at the 6-month follow-up were due to absences at the assessment meeting. The participants' socio-demographics are summarized in Table 2. As observed, the main characteristics were well-balanced across groups, consisting predominantly of young female European university students. There were no differences in any of the clinical variables at baseline (Supplementary Material Table 1).

**Table 2:** Baseline socio-demographic characteristics of participants by treatment group.

**Socio-demographic variables**	**Controls (*n* = 49)**	**Short MBI (*n* = 48)**	**8-Week MBI (*n* = 51)**	***p***
Gender [Females: *n* (%)]	35 (71.4)	32 (66.7)	35 (68.6)	0.878
Mean age, years (*SD*)	21.71 (1.00)	21.56 (3.12)	21.29 (1.77)	0.610
Married/partner, *n* (%)	20 (42.6)	16 (33.3)	21 (41.2)	0.607
No previous meditative experience (previous 6 months), *n* (%)	46 (93.9)	47 (97.9)	48 (94.1)	0.575

There were no significant differences between the abbreviated MBI and the 8-week MBI at the two- and six-month follow-ups in the main outcome (MAAS), nor in the different mindfulness facets such as, observing, describing, acting with awareness, non-judging, and non-reactivity (Table 3). However, there were significant differences in MAAS as well as in all of the mindfulness facets between the control group vs. the abbreviated MBI, and between the control group vs. the 8-week MBI, at post-treatment and the 6-month follow up, with both interventions performing better than the controls. In general, the results with the self-compassion constructs showed no significant differences between groups (Table 4), and significant differences were only found in self-kindness between the abbreviated MBI vs. the 8-week MBI at the 6-month follow-up, in favor of the 8-week MBI group, but with a very small ES (*d* = −0.12).

**Table 3:** Descriptive statistics, effect sizes, and group × time interactions on the mindfulness variables.

	**1. Control group *n* = 47M (*SD*)**	**2. Abbreviated MBP *n* = 46M (*SD*)**	**3. 8-Week MBP *n* = 48M (*SD*)**	***d* 1 vs. 2**	**B (95% CI) 1 vs. 2**	**(*p*) 1 vs. 2**	***d* 1 vs. 3**	**B (95% CI) 1 vs. 3**	**(*p*) 1 vs. 3**	***d* 2 vs. 3**	**B (95% CI) 2 vs. 3**	**(*p*) 2 vs. 3**
**MAAS**												
Pre-treatment	58.61 (11.29)	56.56 (11.75)	56.61 (11.98)									
Post-treatment	57.59 (9.90)	62.67 (11.56)	62.45 (11.22)	−0.47	7.13 (2.44–11.81)	(<0.01)	−0.46	6.86 (2.25–11.48)	(<0.01)	0.02	−0.26 (−4.90–4.38)	(0.91)
6 Months	58.23 (9.84)	64.20 (10.09)	62.94 (11.39)	−0.60	8.50 (3.75–13.24)	(<0.01)	−0.44	6.71 (2.02–11.40)	(<0.01)	0.12	−1.81 (-6.53–2.91)	(0.45)
**FFMQ-OBSERVING**												
Pre-treatment	22.71 (5.36)	25.21 (5.26)	23.76 (4.89)									
Post-treatment	22.33 (5.09)	27.44 (5.26)	26.29 (4.86)	−0.99	2.62 (1–3.67)	(<0.01)	−0.80	2.92 (1.88–3.95)	(<0.01)	0.23	0.30 (−0.33–0.93)	(0.35)
6 Months	21.47 (5.50)	27.65 (5.09)	26.52 (4.67)	−1.17	4.03 (2.96–5.10)	(<0.01)	−0.99	4.16 (3.11–5.22)	(<0.01)	0.23	0.13 (−0.52–0.77)	(0.70)
**FFMQ-DESCRIBING**												
Pre-treatment	27.21 (6.75)	26.02 (7.22)	26.96 (6.02)									
Post-treatment	27.12 (6.38)	28.96 (6.93)	30.55 (6.09)	−0.28	3.03 (2.07–3.99)	(<0.01)	−0.55	3.68 (2.73–4.63)	(<0.01)	−0.24	0.65 (−0.36–1.66)	(0.21)
6 Months	27.21 (6.21)	30.02 (6.87)	31.56 (6.14)	−0.43	3.98 (3.00–4.96)	(<0.01)	−0.70	4.58 (3.62–5.55)	(<0.01)	−0.24	0.60 (−0.43–1.63)	(0.25)
**FFMQ-ACTING**												
Pre-treatment	26.51 (4.91)	25.19 (5.18)	24.86 (5.73)									
Post-treatment	26.65 (4.86)	29.21 (5.36)	29.14 (6.14)	−0.50	3.88 (2.97–4.79)	(<0.01)	−0.45	4.13 (3.23–5.03)	(<0.01)	0.01	0.25 (-0.75–1.26)	(0.62)
6 Months	26.74 (4.90)	30.96 (5.73)	30.63 (5.98)	−0.79	5.70 (4.77–6.62)	(<0.01)	−0.71	5.49 (4.58–6.41)	(<0.01)	0.06	−0.20 (−1.23–0.83)	(0.70)
**FFMQ-NON-JUDGING**												
Pre-treatment	25.94 (6.93)	26.63 (7.79)	26.59 (6.59)									
Post-treatment	25.82 (6.37)	29.79 (7.96)	30.24 (6.52)	−0.55	3.29 (2.31–4.26)	(<0.01)	−0.69	3.77 (2.81–4.73)	(<0.01)	−0.06	0.48 (−0.37–1.33)	(0.27)
6-Months	25.06 (7.38)	29.96 (8.03)	30.63 (6.31)	−0.64	3.63 (2.64–4.62)	(<0.01)	−0.81	4.20 (3.22–5.18)	(<0.01)	−0.09	0.56 (−1.30–1.43)	(0.20)
**FFMQ-NON-REACT**												
Pre-treatment	20.82 (3.71)	21.06 (4.59)	20.29 (3.42)									
Post-treatment	21.35 (3.77)	23.77 (5.21)	23.86 (3.96)	−0.53	2.18 (1.17–3.18)	(<0.01)	−0.65	3.04 (2.05–4.03)	(<0.01)	−0.02	0.86 (−0.06–1.78)	(0.07)
6 Months	21.40 (4.67)	24.52 (5.11)	24.25 (4.13)	−0.64	3.20 (2.18–4.22)	(<0.01)	−0.65	3.66 (2.66–4.67)	(<0.01)	0.06	0.46 (−0.48–1.41)	(0.33)

*M, mean; SD, standard deviation; d, Cohen's d; B, regression coefficients; 95% CI, 95% confidence interval; p, p-values for each comparison*.

**Table 4:** Descriptive statistics, effect sizes, and group × time interactions on the self-compassion variables.

	**1. Control group *n* = 47M (*SD*)**	**2. Abbreviated MBI *n* = 46M (*SD*)**	**3. 8-Week MBI *n* = 48M (*SD*)**	***d* 1 vs. 2**	**B (95% CI) 1 vs. 2**	**(*p*) 1 vs. 2**	***d* 1 vs. 3**	**B (95% CI) 1 vs. 3**	**(*p*) 1 vs. 3**	***d* 2 vs. 3**	**B (95% CI) 2 vs. 3**	**(*p*) 2 vs. 3**
**SCS-SELF-KINDNESS**												
Pre-treatment	2.63 (0.97)	2.82 (1.02)	2.46 (0.72)									
Post-treatment	2.56 (0.95)	2.63 (0.81)	2.56 (0.72)	−0.08	−0.13 (−0.61–0.35)	(0.61)	< 0.01	0.17 (−0.31–0.64)	(0.49)	0.02	0.29 (−0.15–0.74)	(0.19)
6 Months	2.62 (0.92)	2.45 (0.73)	2.72 (0.81)	0.20	−0.36 (−0.85–0.12)	(0.14)	−0.12	0.27 (−0.21–0.75)	(0.27)	−0.12	0.63 (0.18–1.08)	(<0.01)
**SCS-JUDGEMENT**												
Pre-treatment	2.95 (0.92)	2.89 (1.03)	3.12 (0.90)									
Post-treatment	2.84 (0.85)	2.80 (1.06)	2.88 (0.94)	0.04	0.02 (-0.52–0.55)	(0.95)	−0.05	−0.13 (−0.65–0.40)	(0.64)	−0.23	−0.15 (−0.69–0.40)	(0.60)
6 Months	2.89 (0.86)	2.74 (1.03)	2.99 (0.94)	0.16	−0.10 (−0.64–0.45)	(0.73)	−0.11	−0.07 (−0.60–0.46)	(0.79)	−0.23	0.02 (−0.53–0.58)	(0.93)
**SCS-HUMANITY**												
Pre-treatment	2.79 (0.88)	2.65 (0.95)	2.60 (0.77)									
Post-treatment	2.79 (0.84)	2.76 (0.94)	2.53 (0.74)	0.03	0.12 (-0.34–0.58)	(0.61)	0.33	−0.06 (−0.51–0.39)	(0.78)	0.24	−0.18 (−0.65–0.29)	(0.45)
6 Months	2.76 (0.81)	2.77 (0.90)	2.55 (0.87)	−0.01	0.16 (−0.30–0.62)	(0.50)	0.25	−0.03 (−0.48–0.43)	(0.91)	0.24	−0.18 (−0.66–0.30)	(0.46)
**SCS-ISOLATION**												
Pre-treatment	2.51 (1.02)	2.59 (0.99)	2.69 (0.94)									
Post-treatment	2.60 (1.03)	2.71 (0.90)	2.60 (1.01)	−0.11	0.02 (−0.53–0.57)	(0.94)	< 0.01	−0.18 (-0.72–0.36)	(0.52)	0.01	−0.20 (−0.72–0.32)	(0.45)
6 Months	2.62 (1.05)	2.57 (0.97)	2.75 (0.89)	0.05	−0.13 (−0.69–0.43)	(0.65)	−0.13	−0.05 (−0.60–0.50)	(0.85)	−0.06	−0.18 (−0.45–0.61)	(0.77)
**SCS-MINDFULNESS**												
Pre-treatment	2.79 (0.87)	2.90 (0.94)	2.82 (0.78)									
Post-treatment	2.75 (0.82)	2.84 (0.86)	2.78 (0.77)	−0.11	−0.02 (−0.48–0.44)	(0.94)	−0.04	0.01 (−0.45–0.46)	(0.98)	0.06	0.02 (−0.44–0.48)	(0.92)
6 Months	2.70 (0.76)	2.79 (0.90)	2.96 (0.95)	−0.11	−0.01 (−0.48–0.45)	(0.95)	−0.30	0.24 (−0.22–0.70)	(0.30)	−0.09	0.26 (−0.21–0.72)	(0.28)
**SCS-OVERIDENTIFICATION**												
Pre-treatment	3.13 (0.90)	3.17 (0.89)	3.10 (0.88)									
Post-treatment	2.92 (0.94)	3.12 (0.92)	3.17 (0.91)	−0.22	0.16 (−0.36–0.68)	(0.55)	−0.27	0.28 (−0.23–0.79)	(0.29)	−0.02	0.12 (−0.38–0.62)	(0.65)
6 Months	2.81 (1.04)	3.10 (0.91)	3.19 (0.87)	−0.30	0.25 (−0.27–0.77)	(0.35)	−0.40	0.41 (−0.11–0.92)	(0.12)	−0.06	0.17 (−0.35–0.66)	(0.55)

*M, mean; SD, standard deviation; d, Cohen's d; B, regression coefficients; 95% CI, 95% confidence interval; p, p-values for each comparison*.

There were significant differences in PANAS-positive between the control group vs. the abbreviated MBI, and between the control group vs. the 8-week MBI at the 2- and 6-month follow-ups, with both interventions performing better than the controls (Table 5). There were no significant differences between the control group vs. the abbreviated MBI, nor between the control group vs. the 8-week MBI in either PANAS-negative or CD-RISC. There were also no significant differences in HADS-depression between the control group vs. the abbreviated MBI. However, there were significant differences between the control group vs. the 8-week MBI, with lower depression levels in the 8-week MBI group. Significant although marginal differences were observed in HADS-anxiety between the control group vs. the abbreviated MBI. Differences in HADS-anxiety were clear and significant between the control group vs. the 8-week MBI, with higher anxiety levels in the control group. Finally, there were no significant differences between the abbreviated and the 8-week MBI at the two- and six-month follow-ups with regard to PANAS-positive, PANAS-negative, CD-RISC, HADS-anxiety, and HADS-depression.

**Table 5:** Descriptive statistics, effect sizes, and group × time interactions on affects, anxiety, depression, and resilience.

	**1. Control group *n* = 47M (*SD*)**	**2. Abbreviated MBI *n* = 46M (*SD*)**	**3. 8-Week MBI *n* = 48M (*SD*)**	***d* 1 vs. 2**	**B (95% CI) 1 vs. 2**	**(*p*) 1 vs. 2**	***d* 1 vs. 3**	**B (95% CI) 1 vs. 3**	**(*p*) 1 vs. 3**	***d* 2 vs. 3**	**B (95% CI) 2 vs. 3**	**(*p*) 2 vs. 3**
**PANAS-POSITIVE**												
Baseline	35.18 (7.11)	33.46 (7.76)	32.63 (5.61)									
Post-treatment	33.45 (7.52)	40.94 (10.39)	39.29 (8.57)	−0.83	9.21 (5.92–13.30)	(<0.01)	−0.72	8.40 (4.37–12.43)	(<0.01)	0.17	−0.81 (−5.13–3.50)	(0.71)
6 Months	32.23 (6.27)	39.80 (9.16)	41.54 (10.66)	−0.96	9.48 (5.34–13.62)	(<0.01)	−1.07	12.02 (7.94–16.12)	(<0.01)	−0.18	2.54 (−1.84–6.92)	(0.26)
**PANAS-NEGATIVE**												
Baseline	21.96 (6.49)	21.13 (6.26)	21.22 (6.62)									
Post-treatment	21.78 (6.00)	21.10 (6.96)	21.22 (6.34)	0.11	0.16 (−3.39–3.71)	(0.93)	0.09	0.18 (−3.31–3.68)	(0.92)	−0.02	0.02 (-3.46–3.50)	(0.99)
6 Months	22.26 (7.02)	21.09 (6.01)	21.81 (6.77)	0.18	−0.34 (−3.93–3.26)	(0.86)	0.07	0.32 (−3.23–3.86)	(0.86)	−0.11	0.66 (-2.87–4.18)	(0.72)
**HADS-ANXIETY**												
Baseline	7.59 (3.52)	7.04 (3.40)	7.37 (2.81)									
Post-treatment	8.08 (3.18)	6.67 (3.03)	6.55 (2.04)	0.45	−0.87 (−1.72—0.01)	(0.05)	0.57	−1.31 (-2.16—0.47)	(<0.01)	0.05	−0.45 (−1.31–0.41)	(0.31)
6 Months	8.30 (3.11)	6.85 (3.19)	6.73 (2.35)	0.46	−0.91 (−1.78—0.04)	(0.04)	0.57	−1.26 (-2.11—0.40)	(<0.01)	0.04	−0.35 (−1.22–0.53)	(0.43)
**HADS-DEPRESSION**												
Baseline	3.67 (3.04)	3.08 (2.68)	3.31 (2.49)									
Post-treatment	3.67 (2.93)	2.60 (1.94)	2.53 (1.75)	0.43	−0.48 (−1.07–0.11)	(0.11)	0.47	−0.78 (-1.36—0.21)	(<0.01)	0.04	−0.31 (-0.88–0.27)	(0.30)
6 Months	3.91 (3.01)	2.83 (2.03)	2.77 (1.89)	0.42	−0.57 (−1.17–0.02)	(0.06)	0.45	−0.78 (−1.37—0.19)	(<0.01)	0.03	−0.21 (-0.80–0.37)	(0.48)
**CD-RISC**												
Baseline	35.90 (6.36)	35.29 (5.75)	35.39 (5.24)									
Post-treatment	35.31 (6.54)	35.23 (5.82)	35.14 (5.74)	0.01	0.53 (−1.92–2.98)	(0.67)	0.03	0.34 (−2.08–2.75)	(0.78)	0.02	−0.19 (−2.46–2.07)	(0.87)
6 Months	34.68 (5.74)	35.46 (4.74)	35.42 (5.45)	−0.15	1.52 (−0.96–4.01)	(0.23)	−0.13	1.33 (−1.13–3.78)	(0.29)	0.01	−0.20 (−2.50–2.11)	(0.87)

*M, mean; SD, standard deviation; d, Cohen's d; B, regression coefficients; 95% CI, 95% confidence interval; p, p-values for each comparison*.

## Discussion

To the best of our knowledge, this is the first study to directly investigate the efficacy of an 8-week MBI programme and a 4-week abbreviated mindfulness protocol. In our findings, both programmes similarly improved variables that were expected to be sensitive to MBI, such as, mindfulness levels (measured by both MAAS and FFMQ), anxiety, depression, and positive affect (Sears and Kraus, 2009; Strauss et al., 2014; Garland et al., 2015). Compared to controls, the ES of both interventions on those variables was moderate on average, which is similar to previous findings (Khoury et al., 2015). Therefore, both treatment groups performed better than the control group. In fact, judging for the ES obtained, both treatment groups might function equally, but this is just a heuristic hypothesis that will have to be tested adequately in future works by using appropriate non-inferiority designs.

Interestingly, some of the psychological variables assessed showed no change for either intervention. Resilience did not improve, probably because both interventions were too short and unspecific to modify this complex variable in a non-clinical population and because of a possible ceiling effect (Notario-Pacheco et al., 2011). Studies on mindfulness that showed improvements in resilience were either conducted on specifically stressed populations such as, marines (Johnson et al., 2014) or depressed health professionals (Johnson et al., 2015), or the targeted interventions included other specific techniques to improve resilience (Goldhagen et al., 2015).

Differences between the intervention groups in self-kindness, an important component of self-compassion that was included in the 8-week intervention, were found, favoring the 8-week group, which is reasonable since the practice of kindly awareness was included only in it. The other self-compassion measures showed no change. Despite a compassionate attitude being associated with mindfulness itself, the fact that changes in self-compassion may mediate changes in depressive relapse as the result of an MBCT (Kuyken et al., 2010), and the fact that we used a specific practice for compassion (kindly awareness) in our adapted MBI, the programme showed no impact on them. Although some mediation studies observed that compassion did not seem to be a relevant intermediate variable for mindfulness effect in MBSR/MBCT protocols (Gu et al., 2015), one plausible explanation is that the questionnaire itself might have presented some validation problems in Latin European and Latin American populations (Garcia-Campayo et al., 2014; Montero-Marin et al., 2016). PANAS-negative did not improve, probably because of a floor effect, given that PANAS-negative scores in a healthy young population are usually low, and it is difficult for any intervention to cause it to decrease (Garland et al., 2014; Vinci et al., 2014; Witkiewitz et al., 2014). The study was developed using a non-clinical population, with baseline anxiety and depression measures under the cut-off point usually referred for diagnosis. In the case of anxiety, controls were above the referred cut-off at post-treatment and at 6-month follow-up measurements, while the treatment groups stayed below it. In the case of depression, all the groups were below the cut-off point at any time. However, when comparing the treatment groups with the controls, it was observed that the obtained ESs for both outcomes were moderate, with a Cohen's *d* ranging from 0.42 to 0.58, which corresponds to a number needed to treat from 4.28 to 3.14, using the conversion of Kraemer and Kupfer (2006), and therefore is relevant from a clinical point of view.

One remarkable finding is that there were no differences in the effect of an 8-week MBI compared to the abbreviated 4-session programme. To adapt MBIs to real practice—a university environment in this case, in which programmes are intended to be offered freely and in a systematic way to the student population—MBI was administered without the retreat day. In addition, no assessment was made of home practice, intentionally, despite the original MBSR protocol requiring 45 min of home practice per day. In our experience (Demarzo et al., 2014, 2015), this long practice component is not a realistic demand to make on ordinary people in the Latin countries of Europe and the Americas, including university students. Consequently, we did not emphasize it, in order not to discourage the students. Moreover, a more realistic amount of practice (~20 min) is recommended for non-clinical populations (Klatt et al., 2009; Braden et al., 2016; Schroeder et al., 2016). Nevertheless, we are unaware of the amount of home practice that participants performed, so we are limited as to any conclusion in this regard.

Taking these modifications to the standard MBSR programme into consideration, no differences were found between the 8-week MBI and the abbreviated 4-session programme in our study. This can be partly explained because, given the relative scarcity of studies on the efficacy of the components of mindfulness, we do not know which techniques are more effective and should be emphasized and to what extent. Therefore, one possible explanation is that the selection of the practices in the short programme included the most effective ones and followed an appropriate sequence. Another explanation is that the MBSR programme would be unnecessarily long, at least for a healthy, non-clinical population, or for patients with low levels of symptoms (similar to a stepped-care model; Demarzo et al., 2015), and shorter programmes with similar components would probably show similar efficacy. In fact, most of the studies on short mindfulness programmes demonstrate that they are effective at improving some outcomes (Klatt et al., 2009; Braden et al., 2016; Schroeder et al., 2016), particularly psychological distress, and this seems to be independent of the volume of the intervention (number of sessions × session length), and population type (Carmody and Baer, 2009). A similar study, which compared a brief (5-week) mindfulness intervention vs. a parallel control group, found improvements in mindfulness (assessed with the Philadelphia Mindfulness Scale) and self-compassion (measured with the SCS; Bergen-Cico et al., 2013). The ES of the improvements was similar to previous studies that showed MBSR to be moderately effective in reducing stress, depression, anxiety, and distress, and in ameliorating the quality of life of healthy individuals (Khoury et al., 2015). Another alternative explanation might be that participants in the 4-week programme became motivated to undertake a further search for more information about or for longer mindfulness programmes, with the 4-week programme having activated mindfulness behavior in daily life and arousing this motivation. We did not test this hypothesis in our study, but future protocols should perhaps take this into account.

There were a number of limitations in this study. Firstly, some characteristics of the general MBIs (the retreat day and the assessment of home practice) were modified to adapt it to a general population, and these changes may have decreased its efficacy. Secondly, our findings can only be generalized to a population with a high economic and cultural level, such as, that of university students, and we may be unable to extrapolate our findings to other healthy populations or to a clinical population. Moreover, having a predominantly female sample may have had an impact on the obtained outcomes, which suggests that there is a need to evaluate this issue in more detail in future research. Although the groups did not differ in the outcome measures at baseline, given the non-randomization of the control group, this group may have suffered from selection bias, differing from the other two groups with regard to some important variables (e.g., treatment expectations, social desirability bias, and willingness to learn mindfulness), which may have inflated the results of the study conditions. Thirdly, this study was not powered to detect small differences between the two intervention groups (the *post-hoc* statistical power for the MAAS when comparing both intervention groups was 7%, which is too low and could drive to a type-II error); therefore, this aspect of the study needs to be tested adequately in future research (for instance, using a non-inferiority design). Finally, in addition to the fact that we did not systematically evaluate formal practice, all outcome measures were self-related psychological questionnaires, and some outcomes may have been biased or overvalued, as has been demonstrated in some mindfulness measures. For instance, it has been demonstrated that the FFMQ “acting with awareness” subscale did not show adequate reliability and that the “observing” subscale does not seem to be adequate for assessing mindfulness in individuals without meditative practice (Aguado et al., 2015).

In conclusion, both standard 8-week and abbreviated 4-week mindfulness programmes seem to be efficacious for promoting well-being, although many questions remained unanswered, such as, the minimum number and length of sessions and the daily practice required to maintain effectiveness, the kind and order of the therapeutic components and mindfulness exercises to be included, and the characteristics of the participants (individual differences) that might predict better results. Future studies should confirm our findings with more powerful designs to identify small differences between groups, calculate the minimum efficacious dose (number of sessions/hours), and identify the most effective practices and the best sequence in which to administer them in order to develop the shortest and most effective programme available and to make them more accessible for a larger number of clinical and non-clinical populations.

## Ethics statement

The study protocol was approved by the ethical review board of the regional health authority of Aragon, Spain (PI12/00134).

## Author contributions

MD, SM, JM, MP, PH, and JG conceived and designed the study. MD administered Mindfulness Interventions. JG checked treatment integrity. MP, MN, and PH supervised the collection of data. JM analyzed and interpreted the data. MD, JM, SM, JG, SC, and LB produced the drafting of the manuscript. MD and JG supervised all steps in the study. All authors provided a critical revision of the manuscript.

### Conflict of interest statement

The authors declare that the research was conducted in the absence of any commercial or financial relationships that could be construed as a potential conflict of interest.
