# Supplementary material for: Efficacy of 8- and 4-Session Mindfulness-Based Interventions in a Non-clinical Population: A Controlled Study
Source: Front Psychol. 2017 Aug 8;8:1343. doi: 10.3389/fpsyg.2017.01343 (PMC5550824; doi:10.3389/fpsyg.2017.01343)
Supplement: Supplementary file 1 [file Table1.DOCX]

**Supplementary material Table 1:**

**One-way analyses of variance by group at baseline**

| **Outcomes** | **Control**  **Group (n=49)**  **Mn (SD)** | **Abbreviated MBSR (n=48)**  **Mn (SD)** | **Standard**  **MBSR (n=51)**  **Mn (SD)** | **F (d.f.)** | **p** |
| --- | --- | --- | --- | --- | --- |
| MAAS | 58.61 (11.29) | 56.56 (11.75) | 56.61 (11.98) | 0.49 (2, 145) | 0.612 |
|  |  |  |  |  |  |
| FFMQ-observing | 22.71 (5.36) | 25.21 (5.26) | 23.76 (4.89) | 2.84 (2, 145) | 0.062 |
|  |  |  |  |  |  |
| FFMQ-describing | 27.21 (6.75) | 26.02 (7.22) | 26.96 (6.02) | 0.43 (2, 145) | 0.651 |
|  |  |  |  |  |  |
| FFMQ-acting | 26.51 (4.91) | 25.19 (5.18) | 24.86 (5.73) | 1.35 (2, 145) | 0.264 |
|  |  |  |  |  |  |
| FFMQ-nonjudging | 25.94 (6.93) | 26.63 (7.79) | 26.59 (6.59) | 0.15 (2, 145) | 0.865 |
|  |  |  |  |  |  |
| FFMQ-nonreact | 20.82 (3.71) | 21.06 (4.59) | 20.29 (3.42) | 0.50 (2, 145) | 0.609 |
|  |  |  |  |  |  |
| SCS-self-kindness | 2.63 (0.97) | 2.82 (1.02) | 2.46 (0.72) | 1.97 (2, 145) | 0.143 |
|  |  |  |  |  |  |
| SCS-judgement | 2.95 (0.92) | 2.89 (1.03) | 3.12 (0.90) | 0.80 (2, 145) | 0.453 |
|  |  |  |  |  |  |
| SCS-humanity | 2.79 (0.88) | 2.65 (0.95) | 2.60 (0.77) | 0.64 (2, 145) | 0.530 |
|  |  |  |  |  |  |
| SCS-isolation | 2.51 (1.02) | 2.59 (0.99) | 2.69 (0.94) | 0.43 (2, 145) | 0.652 |
|  |  |  |  |  |  |
| SCS-mindfulness | 2.79 (0.87) | 2.90 (0.94) | 2.82 (0.78) | 0.19 (2, 145) | 0.825 |
|  |  |  |  |  |  |
| SCS-overidentification | 3.13 (0.90) | 3.17 (0.89) | 3.10 (0.88) | 0.09 (2, 145) | 0.917 |
|  |  |  |  |  |  |
| PANAS-positive | 35.18 (7.11) | 33.46 (7.76) | 32.63 (5.61) | 1.80 (2, 145) | 0.170 |
|  |  |  |  |  |  |
| PANAS-negative | 21.96 (6.49) | 21.13 (6.26) | 21.22 (6.62) | 0.25 (2, 145) | 0.782 |
|  |  |  |  |  |  |
| HADS-anxiety | 7.59 (3.52) | 7.04 (3.40) | 7.37 (2.81) | 0.35 (2, 145) | 0.705 |
|  |  |  |  |  |  |
| HADS-depression | 3.67 (3.04) | 3.08 (2.68) | 3.31 (2.49) | 0.57 (2, 145) | 0.566 |
|  |  |  |  |  |  |
| CD-RISC | 35.90 (6.36) | 35.29 (5.75) | 35.39 (5.24) | 0.15 (2, 145) | 0.858 |

Mn = mean. SD = standard deviation. F (d.f.) = Snedecor’s F (degrees of freedom). p = p-value.
